# A Modified Hyaluronic Acid–Based Dissolving Microneedle Loaded With Daphnetin Improved the Treatment of Psoriasis

**DOI:** 10.3389/fbioe.2022.900274

**Published:** 2022-06-17

**Authors:** Shiya Peng, Liuhanghang Cheng, Qian Wu, Yuanchao Li, Lei Ran, Wei Wang, Ke Huang, Rong Zhu, Sihong Xue, Chunli Zhou, Weidong Zhu, Biao Cheng, Xiaobing Fu, Rupeng Wang

**Affiliations:** ^1^ Department of Dermatology and Rheumatology Immunology, Xinqiao Hospital, Third Military Medical University (Army Medical University), Chongqing, China; ^2^ Research Center for Tissue Repair and Regeneration Affiliated to the Medical Innovation Research Department and Fourth Medical Center, PLA General Hospital and PLA Medical College, Beijing, China; ^3^ PLA Key Laboratory of Tissue Repair and Regenerative Medicine and Beijing Key Research Laboratory of Skin Injury, Repair and Regeneration, Beijing, China; ^4^ Research Unit of Trauma Care, Tissue Repair and Regeneration, Chinese Academy of Medical Sciences, Beijing, China; ^5^ The First School of Clinical Medicine, Southern Medical University, Guangzhou, China; ^6^ Department of Burn and Plastic Surgery, General Hospital of Southern Theater Command, Guangzhou, China

**Keywords:** psoriasis, daphnetin, hyaluronic acid microneedles, inflammatory factor, CCL20

## Abstract

Psoriasis is a common chronic immune-inflammatory disease. Challenges exist in the present treatment of psoriasis, such as difficulties in transdermal drug administration and severe side effects. We hope to achieve a better therapeutic outcome for psoriasis treatment. By using modified soluble microneedles (MNs) loaded with daphnetin, the psoriasis symptoms of mice, the abnormal proliferation of keratinocytes, and the secretion of inflammatory factors were significantly reduced. *In vitro*, daphnetin is proven to inhibit the NF-κB signaling pathway and to inhibit the proliferation of HaCaT cells and the release of inflammatory factors, especially CCL20. This research showed that the modified microneedle loaded with daphnetin optimized transdermal drug delivery and relieved the symptoms of psoriasis more effectively. The novel route of Daph administration provides a future research direction for the treatment of psoriasis.

## 1 Introduction

Characterized by its skin erythema and scales symptomatically and hyperkeratosis with dyskeratosis and massive lymphocyte infiltration pathologically, psoriasis, as a common chronic immune disease, has affected over 125 million people worldwide (Nestle et al., 2009; Lowes et al., 2014; Armstrong and Read, 2020; Vicic et al., 2021). Skin damage is triggered and aggravated by the Koebner phenomenon (Weiss et al., 2002). The progression of psoriasis is associated with a variety of cells types, including dendritic cells, neutrophils, endothelial cells, fibroblasts, keratinocytes, and lymphocytes, among which, keratinocytes and T cells play an important role in the inflammatory response of psoriasis (Georgescu et al., 2019; Shin et al., 2020). Keratinocytes proliferate abnormally under the stimulation of many factors and secrete a series of chemokines such as CXCL1, CXCL2, CXCL8, and CCL20 (8). These chemokines recruit a variety of inflammatory cells to infiltrate locally and produce IL-1β, IL-23, TNF-α, and other inflammatory factors with keratinocytes to amplify the inflammatory response of psoriasis (Albanesi et al., 2018). T cells, especially Vγ4+ T cells, play a key role among the recruited inflammatory cells (Zhu et al., 2017). CCR6-expressing Vγ4+ T cells can be recruited by CCL20 and secrete IL-17A, IL-22, and IFN-γ in response to IL-1β, IL-23, and TNF-α (Homey et al., 2000). IL-17A, IL-22, and IFN-γ can promote the abnormal proliferation of keratinocytes to form a positive feedback loop (Furue et al., 2020a) and continuously aggravate the inflammatory response of psoriasis. The clearance of CCL20 has been proven to significantly decrease the infiltration of γδ T cells in mice and relieve the symptoms of psoriasis (Mabuchi et al., 2013). Therefore, reducing the release of inflammatory factors associated with epidermal cells and inflammatory cells is one of the important therapeutic concepts (Kim et al., 2021).

Daphnetin (7,8-dihydroxycoumarin) is isolated from Daphne Korean Nakai and used for cancer and inflammatory disease treatment because of its antiproliferation and anti-inflammation effects (Pei et al., 2021). Previous research has shown the therapeutic effectiveness of Daph (in a cream form) in a psoriasis mouse model (Gao et al., 2020). Daph was reported to inhibit the abnormal proliferation of keratinocytes by inhibiting the NF-κB signaling pathway, thus reducing the release of inflammatory factors and alleviating the symptoms of psoriasis. The effect of Daph on Vγ4+ T cells, however, has not been studied, and the expression of CCL-20, a key chemokine in the inflammatory ring between keratinocytes and Vγ4+ T cells, was not measured. In addition, in previous studies, Daph could only inhibit and improve psoriasis lesions and did not show a complete cure effect, so its therapeutic effect needs to be further improved.

Despite the acceptable therapeutic effectiveness of Daph cream in psoriasis, even and continuous intradermal drug distribution is expected. The thickened stratum corneum in psoriasis also affects the transdermal absorption of topical drugs. Therefore, instead of cream, we chose hyaluronic acid (HA) microneedles (MNs), which have been proven in many studies to be effective in intralesional drug delivery (Huang et al., 2020; Liu et al., 2020). In prior studies, hyaluronic acid–based soluble MN patches loaded with methotrexate for intralesional administration have also been shown to improve the therapeutic efficacy in psoriasis and reduce the side effects compared with oral administration (Du et al., 2019). However, there is no relevant study on the topic of soluble MNs loaded with Daph in psoriasis. In addition, we have improved the MNs by adding polyvinyl acetate (PVA) as a matrix and cellulose nanocrystals (CNCs) to enhance the strength of the needle tip.

In this study, we use the modified MN combined with Daph as described previously to improve the treatment of psoriasis at a lower drug dosage. The changes in keratinocytes and Vγ4+ T cells and the expression of key inflammatory factors released by them were detected *in vivo*. *In vitro*, we used mRNA sequencing and other techniques to comprehensively detect the effect of Daph on HaCaT cells and on the release of CCL-20 and the mechanism.

## 2 Materials and Methods

### 2.1 Preparation of the HA/PVA/CNC Patches

First, 2 g CNC was dissolved in 18 ml 45% concentrated sulfuric acid and reacted in a water bath at 45°C for 6 h. The reaction was stopped with 200 ml of deionized water, and the suspension was centrifuged (3000 r/min). A dialysis bag was used for dialysis for 3 days until the pH of the suspension was close to neutral, and the suspension was stored at −20°C. The suspension was then lyophilized at - 80°C to obtain CNC powder. Daph was dissolved in absolute ethanol (0.01%, w/v). For the preparation of the Daph-loaded MN patch, Daph solution was added to the dimethylsiloxane (PDMS) mold and bubbles were removed in a vacuum oven. CNC powder was dissolved in deionized water at room temperature (0.1%, w/v) and then placed in the PDMS mold and bubbles were removed in a vacuum oven. After the CNC was dried, HA solution was placed in the PDMS mold, and the bubbles were removed in a vacuum oven. The excess HA solution was removed, and PVA solution was added to the PDMS mold and the bubbles were removed in a vacuum oven. The PDMS mold was placed in the dark at room temperature for 4 days, and PVA solution was added four times during the process. Finally, the tips of the needle consisted of 30% HA and 0.1% CNC, and the substrate consisted of 30% PVA (Supplementary Figure S1).

### 2.2 Properties of the Microneedle Patches

#### 2.2.1 Infrared Spectroscopy

Three–five mg CNC sample and potassium bromide powder are fully ground and mixed in a mortar. An appropriate amount of powder was pressed into tablets (20 mmHg, 5 min) and detected by using a Fourier infrared spectrometer (Bruker, Germany) at 4000–500 cm-1.

#### 2.2.2 X-Ray Diffraction Pattern of Cellulose Nanocrystal

Rheological properties were detected by a 20-mm diameter stainless steel parallel plate swivel (Bruker, Germany). G′ represented the elastic modulus of the samples, and G″ represented the viscous modulus. At room temperature, the fixed strain was 1% and time scanning was performed and G′ and G″ were recorded. Dynamic strain scanning was then performed at room temperature from 0.1 to 1000 Hz to determine the linear viscoelastic range of hydrogels.

#### 2.2.3 Cellulose Nanocrystal Particle Size Determination

The particle size of the CNC was determined by a Zeta Sizer Nano ZS (Malvern, England).

#### 2.2.4 Transmission Electron Microscopy Characterization of Cellulose Nanocrystal

The microscopic characterization of the CNC was determined by transmission electron microscopy (Hitachi, Japan).

#### 2.2.5 Appearance of the Microneedle Patches

The MN patch was put on an Mshot stereomicroscope (Mingmei, China), and the tip area of the patches was photographed. The freeze-dried patches were pasted on the copper table with carbon conductive adhesive and observed by using a scanning electron microscope (Philips, Netherlands).

#### 2.2.6 Tip Strength of the Microneedle Patches

The MN patch was put on the sample table of a universal material testing machine (BOSE, United States). The tip of the patch was up and compressed to 0.3 mm at a constant rate of 0.01 mm/s. The single-needle strength was calculated from the force recorded by the instrument, and the shape changes in the needle tip after compression were photographed by using a digital camera.

#### 2.2.7 *In Vivo* Biocompatibility of the Microneedle Patches

The patches were pressed on the skin of nude mice for 5 min. The changes in the acupuncture area were observed at different time points and photos were taken.

### 2.3 *In Vivo* Puncture and Drug Release of the HA/PVA/CNC Patches

#### 2.3.1 Penetration Test

The patch was pressed on isolated swine skin for 2 min, and then the tissue was fixed with 4% paraformaldehyde, embedded in paraffin, and sectioned. After hematoxylin and eosin (H&E) staining, the penetration was observed under a microscope.

#### 2.3.2 Puncture Dissolution Test of the Tips

The patches were pressed on the back skin of nude mice and removed after 10 s, 30 s, 1, 2, and 5 min. The dissolution of the tips before and after puncture was observed and photographed under a light microscope.

#### 2.3.3 Fluorescence Confocal Drug Permeation Test

Hydrophobic adriamycin was loaded onto the needle tip as a model fluorescent molecule since Daph was insoluble in water and its fluorescence was weak. After 0.5 h of swine skin puncture, the fluorescence penetration depth of needle tip drugs was scanned by using a laser confocal microscope (Zeiss, Germany).

### 2.4 Animals and Psoriasis-like Inflammation Model

The female C57/BL6 mice were obtained from the Experimental Animal Center of the Army Medical University and kept at 22–24°C and 50% humidity. All the mice had free access to food and water, and every mouse was kept in a cage.

All the mice were anesthetized with 1% pentobarbital solution (Sigma, United States), and a 2.5*3 cm area was selected on the back of the mouse and shaved carefully. Fifteen mice were randomized into five groups: control group (normal group), imiquimod (IMQ) group, Daph group, vehicle group, and vehicle + Daph group. Each mouse was applied with 62.5 mg IMQ ointment on the prepared back skin once daily, except for the control group. Gauze containing Daph (0.1 mg) was applied on the molding area of the Daph group and renewed every day. The vehicle group and vehicle + Daph group were treated once daily with Daph patches and Daph-free patches, respectively. Photos of the experimental skin area of all the mice were taken once a day, and all mice were killed on the seventh day for follow-up detection.

### 2.5 Psoriasis Area Severity Index (PASI) Score and Histological Analysis

The erythema and scales of mice in different groups were scored by the psoriasis area severity index (PASI) (41): 0, none; 1, slight; 2, moderate; 3, severe; and 4, very severe. The skin on the back modeling area was fixed with 4% paraformaldehyde dehydrated, paraffin-embedded, and made into 5-μm paraffin sections. Hematoxylin (Beyotime, China) and eosin (H&E) staining was performed for sections of each group. All sections were observed and counted under an optical microscope. Image-pro Plus 6.0 was used for the calculation of the epithelial thickness of the skin.

### 2.6 Immunohistochemistry and Immunofluorescence Staining

Gradient concentrations of xylene and alcohol were used for paraffin section dewaxing and hydration, and then the sections were heated for antigen retrieval. After cooling to room temperature, the sections were soaked in 3% H_2_O_2_ solution to inactivate the endogenous peroxidase (only for immunohistochemistry). All sections were treated with 10% goat serum for 30 min to block the antigen and then incubated with primary antibodies at 4°C overnight. The secondary antibody (Zhongshan Biology Co. Ltd, China) corresponding to the primary antibody was incubated at room temperature for 30 min. For immunohistochemistry staining, diaminobenzidine was used for antibody staining and hematoxylin (Beyotime, China) for nuclear staining. For immunofluorescence staining, DAPI was used for nuclear staining. The results were observed and photographed under a microscope (Olympus, Japan).

The primary antibodies used are described as follows: IL-1β (1:200, Abcam, United Kingdom); IL-6 (1:200, Abcam, United Kingdom); IL-8 (1:200, Bioworld, United States); K1 (1:200, Abcam, United Kingdom); K6 (1:200, Thermo Fisher Scientific, United States); TNF-α (1:200, Abcam, United Kingdom); IL-23 (1:200, Abcam, United Kingdom); CCL20 (1:100, Abcam, United Kingdom); IL-22; IL-17 (1:250, Abcam, United Kingdom); IFN-γ (1:200, Abcam, United Kingdom); and BrdU (Abcam, United Kingdom).

### 2.7 Isolation of Dermal Cells and Flow Cytometry

Skin samples were obtained from the back modeling area and the subcutaneous fascia was scraped off, then treated with 0.5% trypsin/GNK solution at 37°C for 2 h. After that, the dermis and epidermis were separated, The separated dermis and epidermis were cut into particles and then digested at 37°C for 1 h. Finally, the single-cell suspension was prepared by filtration with a 70-μm screen.

The single-cell suspensions were stained with monoclonal flow cytometry antibodies. For cell surface marker staining, anti CD16/32 was used for the nonspecific binding site and incubated with antibodies for 30 min at room temperature. For intracellular markers, the cells were incubated with a cocktail for 4 h and then stained for surface markers. After that, the cells were fixed with BD Cytofix Buffer and permeabilized with a Perm/Wash reagent. Finally, the cells were stained by intracellular antibodies and detected by using an Attune Acoustic Focusing Cytometer (Attune, Applied Biosystems AB, United States). The data were analyzed by Flowjo software.

The antibody used is described as follows: CD3 (BD Bioscience, United States); Vγ4 (BD Bioscience, United States); and IL-17 (BD Bioscience, United States).

### 2.8 Cell Culture and Biological Functions

Human HaCaT cells (Chinese Academy of Sciences, China) were cultured in a 5% CO_2_ incubator at 37°C. The cells were passaged when they covered about 70% area of the culture bottle. The corresponding stimulation was performed as follows: the control group was cultured by a completed DMEM; the IMQ group was stimulated by 10 μg/ml IMQ; the Daph group was stimulated by 20 μM Daph; and the IMQ + Daph group was stimulated by both 10 μg/ml IMQ and 20 μM Daph.

#### 2.8.1 Cytotoxicity

Cytotoxicity was detected by the CCK-8 method. The HaCaT cell suspension was cultured in a 96-well plate (2,000 cells/well). After cell adhesion, stimulating factors were added to the medium as mentioned previously. A total of 10 μL CCK-8 was added to the well after 0, 12, 24, 48, and 72 h and incubated at 37°C for 4 h. An OD value of 450 nm was detected by a microplate reader.

#### 2.8.2 Cell Proliferation

An Edu test kit (Invitrogen, United States) was used for the detection of cell proliferation. According to the instruction, 5 μmol/L Edu was added into a well 3 h before the cells were stained. Finally, the result was detected by using a flow cytometer.

#### 2.8.3 Apoptosis

HaCaT cells were collected after grouping stimulation was performed as described previously and washed in PBS. The cells were resuspended in 200 μL binding buffer (1×) and then treated according to the instruction of an Annexin V-FITC apoptosis kit (Thermo Fisher Scientific, United States). The result was detected by using a flow cytometer.

### 2.9 RNA Contraction, Quantitative Real-Time PCR (qPCR), and Western Blot

For the skin sample, the tissue was ground in liquid nitrogen, and then the RNA was isolated by TRIzol reagent (Ambion Life Technologies, United States). For the cells, RNA was extracted by using TRIzol directly. After the quantification of RNA concentration, cDNA was synthesized by using a reverse transcription kit (Takara, Japan). The cDNA was treated according to the instruction of the qPCR kit, and the result was detected by using an instrument (CFX Connect™, BIO-RAD, United States).

Primers:

Mouse:

β-actin:5′- AGAGGGAAATCGTGCGTGAC-3′(forward)

5′- CAATAGTGATGACCTGGCCGT-3′(reverse)

IL-6:5′- ACAGAAGGAGTGGCTAAGGA-3′(forward)

5′- AGGCATAACGCACTAGGTTT-3′(reverse)

IL-23:5′- ACCAAAGGAGGTGGATAGG-3′(forward)

5′- GGCAACAGCCATAGCATT-3′(reverse)

IL-1β:5′- AGTTGACGGACCCCAAA-3′(forward)

5′- TCTTGTTGATGTGCTGCTG-3′(reverse)

IL-22:5′- ATCTCTGATGGCTGTCCTG-3′(forward)

5′- GTATGGCTGCTGGAAGTTG-3′(reverse)

TNF-α:5′- CGCTGAGGTCAATCTGC-3′(forward)

5′- GGCTGGGTAGAGAATGGA-3′(reverse)

IL-17:5′- TCCAGGGAGAGCTTCATCTGTGTC-3′(forward)

5′- TTGGACACGCTGAGCTTTGAGG-3′(reverse)

KRT1:5′- GACTCGCTGAAGAGTGACCAGT-3′(forward)

5′- GGTCACGAACTCATTCTCTGCG-3′(reverse)

KRT6:5′- CTGTGAGTTTCTAATGGCCTGAGA-3′(forward)

5′- GAAACTTACATCACAGGACCAGTGA-3′(reverse)

IFN-γ:5′- CAGCAACAGCAAGGCGAAAAAGG-3′(forward)

5′- TTTCCGCTTCCTGAGGCTGGAT-3′(reverse)

CXCL1:5′- CTGGGATTCACCTCAAGAACATC-3′(forward)

5′- CAGGGTCAAGGCAAGCCTC-3′(reverse)

CXCL8:5′- CTTTGTCCATTCCCACTTCTGA-3′(forward)

5′- TCCCTAACGGTTGCCTTTGTAT-3′(reverse)

Human:

GAPDH:5′- TGTTGCCATCAATGACCCCTT-3′(forward)

5′- CTCCACGACGTACTCAGCG-3′(reverse)

CCL20:5′-TGCTGTACCAAGAGTTTGCTC-3′(forward)

5′- CGCACACAGACAACTTTTTCTTT-3′(reverse)

IL-6:5′- ACTCACCTCTTCAGAACGAATTG-3′(forward)

5′- CCATCTTTGGAAGGTTCAGGTTG-3′(reverse)

IL-8:5′- ACTGAGAGTGATTGAGAGTGGAC-3′(forward)

5′- AACCCTCTGCACCCAGTTTTC-3′(reverse)

TNF-α:5′- CCTCTCTCTAATCAGCCCTCTG-3′(forward)

5′- GAG​GAC​CTG​GGA​GTA​GAT​GAG -3′(reverse)

K6:5′- GGGTTTCAGTGCCAACTCAG-3′(forward)

5′- CCA​GGC​CAT​ACA​GAC​TGC​GG -3′(reverse)

#### 2.9.1 Western Blot

The protein of HaCaT cells was extracted by using RIPA lysis buffer (Beyotime, China) with protease and phosphatase inhibitors. A BCA protein assay kit (Thermo Fisher Scientific, United States) was used for the determination of protein concentration, and standard samples were prepared by boiling. The protein samples were passed through SDS-polyacrylamide gel electrophoresis and then transferred to the PVDF membrane. The membrane was blocked in 5% BSA and incubated with a primary antibody at 4°C overnight. After that, a secondary antibody was incubated at room temperature for 1 h. Finally, the membrane was incubated with a chemiluminescence substrate and visualized by the ChemiDoc™ XRS + system.

The antibody used is described as follows: CCL20 (1:1000, Abcam, United Kingdom); anti-p65 (1:1000, CST, United States); and -p-p65 (1:1000, CST, United States).

### 2.10 Ethics Approval

The animal study was reviewed and approved by the Medical and Ethics Committee of the Army Medical University, Chongqing, China.

### 2.11 Statistics Analysis

All data were analyzed by GraphPad Prism 5.0 and presented as the mean ± SD. *p* value <0.05 was considered statistically significant.

## 3 Results

### 3.1 Cellulose Nanocrystals Improved the Shape and Mechanical Properties of Microneedles

**FIGURE 1:**
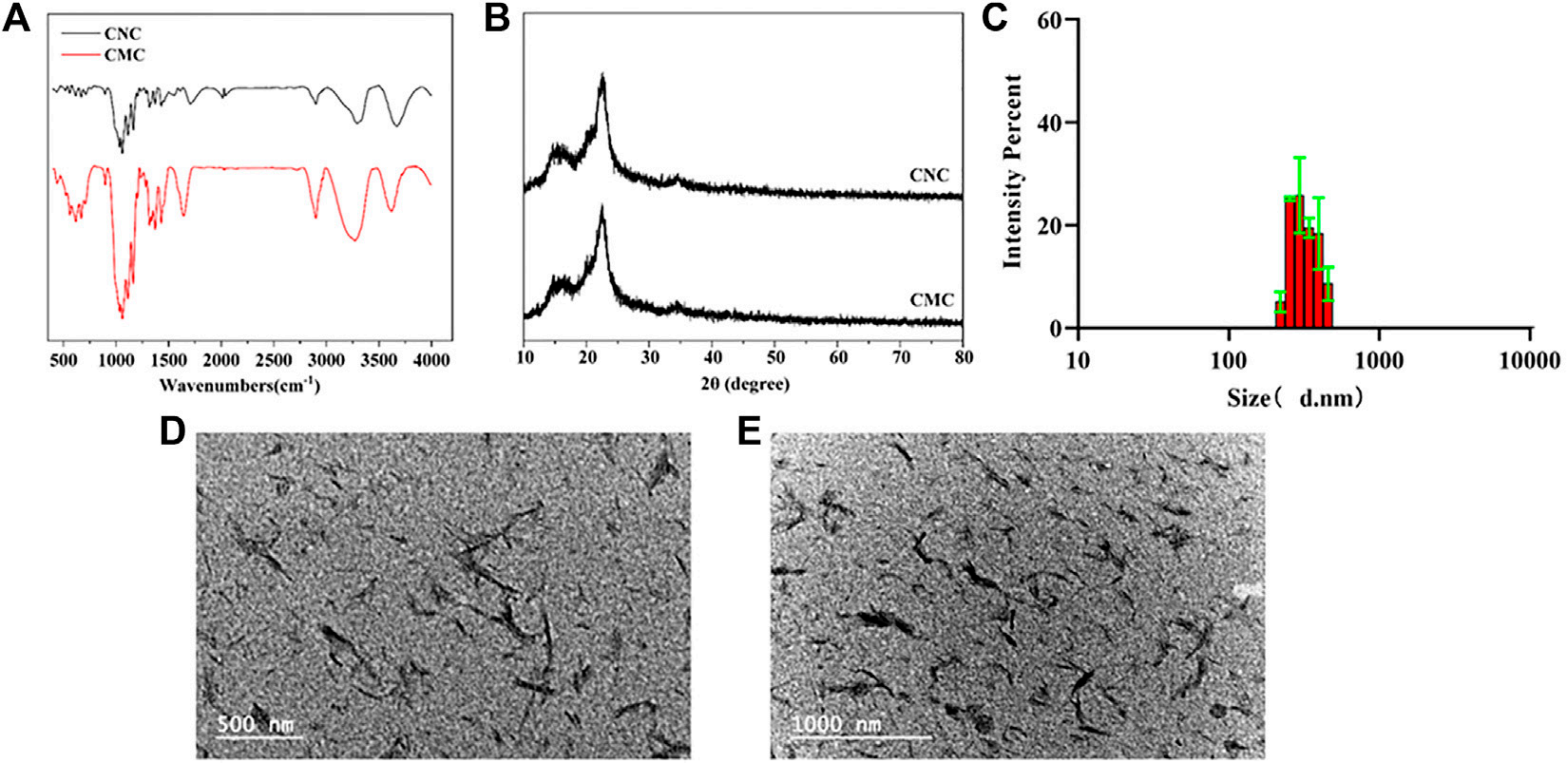
Modification and characterization of the CNC: **(A)** Fourier transform infrared spectra of the CNC and CMC; **(B)** X-ray diffraction patterns of the CNC and CMC; **(C)** DLS image of the CNC; and **(D,E)** TEM images of the CNC at different magnifications. Data are shown as mean ± SE (*p < 0.05; **p < 0.01).

According to the results of the Fourier transform infrared spectrum of the CNC, the peaks at different positions represent the activities of different groups: the peaks at 3431 cm-1 and 1643 cm-1 represented stretching vibration and bending vibration, respectively, of the hydroxyl group (-OH) of cellulose; the peaks at 2901 cm-1 and 1371 cm-1 represented the stretching vibration and bending vibration, respectively, of CH; the peaks at 1016 cm-1 was attributed to the -CO- structure stretching vibration; the peaks at 1159-1 and 1111-1- were typical peaks of cellulose fingerprints; the peaks at 1277 cm-1 and 821 cm-1 were generated by sulfate groups of sulfuric acid (Figure 1A). XRD results showed that crystallization peaks appeared at 16.1°, 22.6°, and 34.6° of 2θ, which meant that the crystal structure of cellulose I can be maintained after acid hydrolysis. In particular, the XRD pattern of the CNC appeared at two new diffraction peaks, at 12.9° and 20.2° of 2θ, indicating the coexistence of cellulose I and cellulose II (Figure 1B). The average particle size of the prepared CNC is 220.2–458.7 nm (Figure 1C), and the CNC is evenly dispersed in a solution with homogeneous particle size (Figure 1D,E).

**FIGURE 2:**
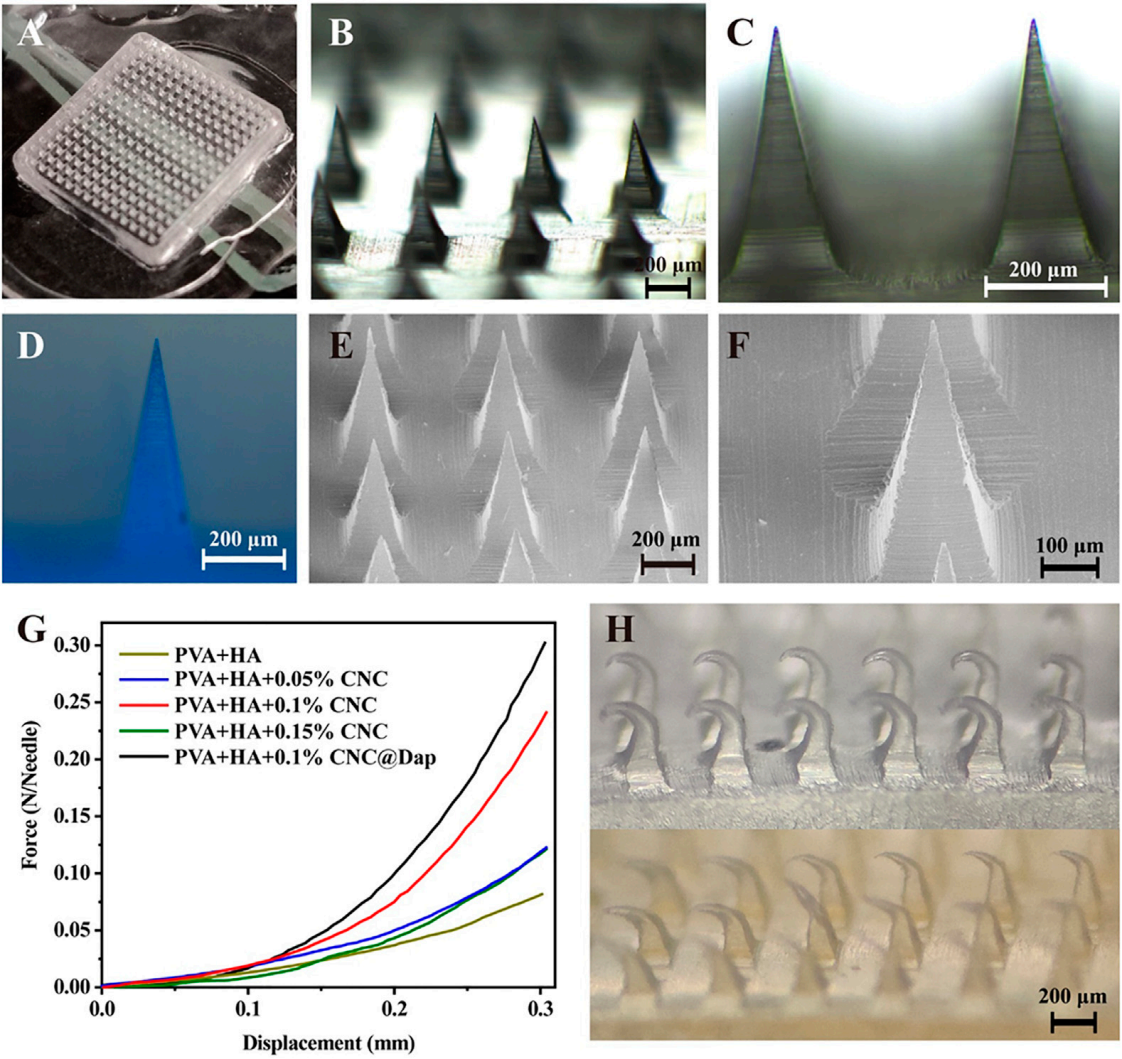
Morphology and mechanical properties of the MN were determined. **(A–C)** Morphology of a blank MN under different magnification of an optical microscope. **(D)** Fluorescence image of a MN loaded with Daph at a scale of 200 μm. **(E, F)** Images of an MN under SEM at scales of 100 and 200 μm. **(G, H)** Test results of mechanical properties of the MN and optical microscope images of a blank MN (above) and drug-loaded MN (below) after compression, the microneedles in the blank and drug loading groups had corresponding morphological changes **(H)**.

The prepared MN was observed by using a light microscope, fluorescence microscope, and scanning electron microscope. The blank MN without drug loading appeared with a 15 × 15 mm array (Figure 2A) and the single needle appeared as a transparent pyramid, of which the height was 600 μm and weight was 200 μm under a light microscope (Figure 2B–F). The surface of the MN was observed to be smooth and complete under the scanning electron microscope. According to the result, when 0.1% CNC is loaded, the force borne by a single MN was maximum, which could reach 0.23 N (Figure 2G). After compression, the microneedles in the blank and drug loading groups had corresponding morphological changes (Figure 2H).

### 3.2 Modified Microneedle Exhibited Good Drug Carriage and Subcutaneous Drug Delivery

**FIGURE 3:**
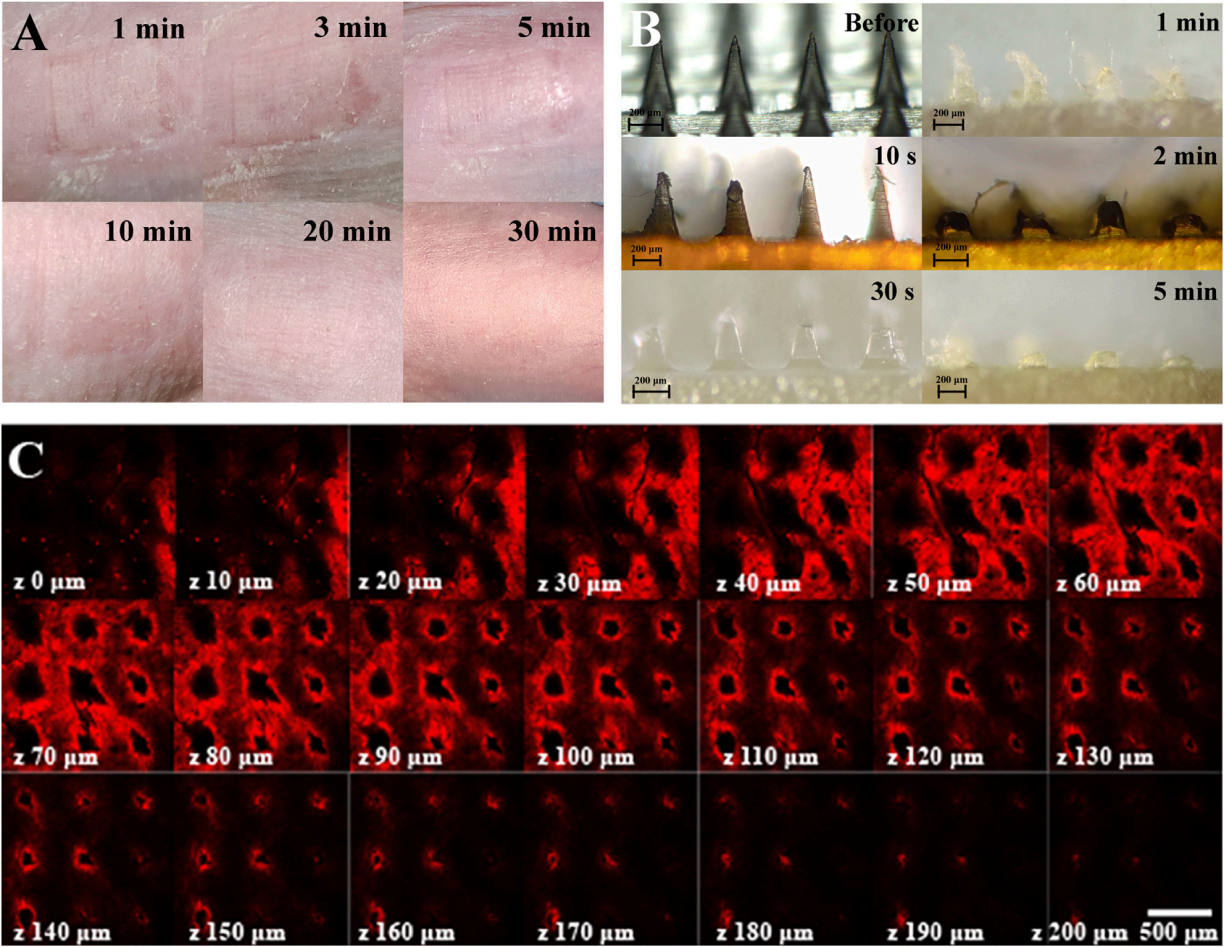
Skin puncture test and drug release characterization of an MN. **(A)** Recovery of the skin puncture site *in vivo.* **(B)** Dissolved state of the MN in pig skin punctured by the MN at different time points. **(C)** Laser confocal image of drug penetration depth of fluorescein-loaded, MN-pierced pig skin, the scale is 500 μm.

The *in vitro* skin puncture experiment of nude mice showed that the skin could recover completely after 30 min of an MN patch puncture (Figure 3A). The MN was pressed on the back skin of nude mice and removed after 10 s, 30 s, 1, 2, and 5 min. The MN was observed to be dissolved completely after 5 min (Figure 3B), and the depth of delivery could be over 200 μm, that is, the subcutaneous layer (Figure 3C).

### 3.3 Daph-Loaded Microneedle Patch Could Alleviate the Symptoms of Psoriasis-Like Dermatitis Induced by IMQ in Mice

**FIGURE 4:**
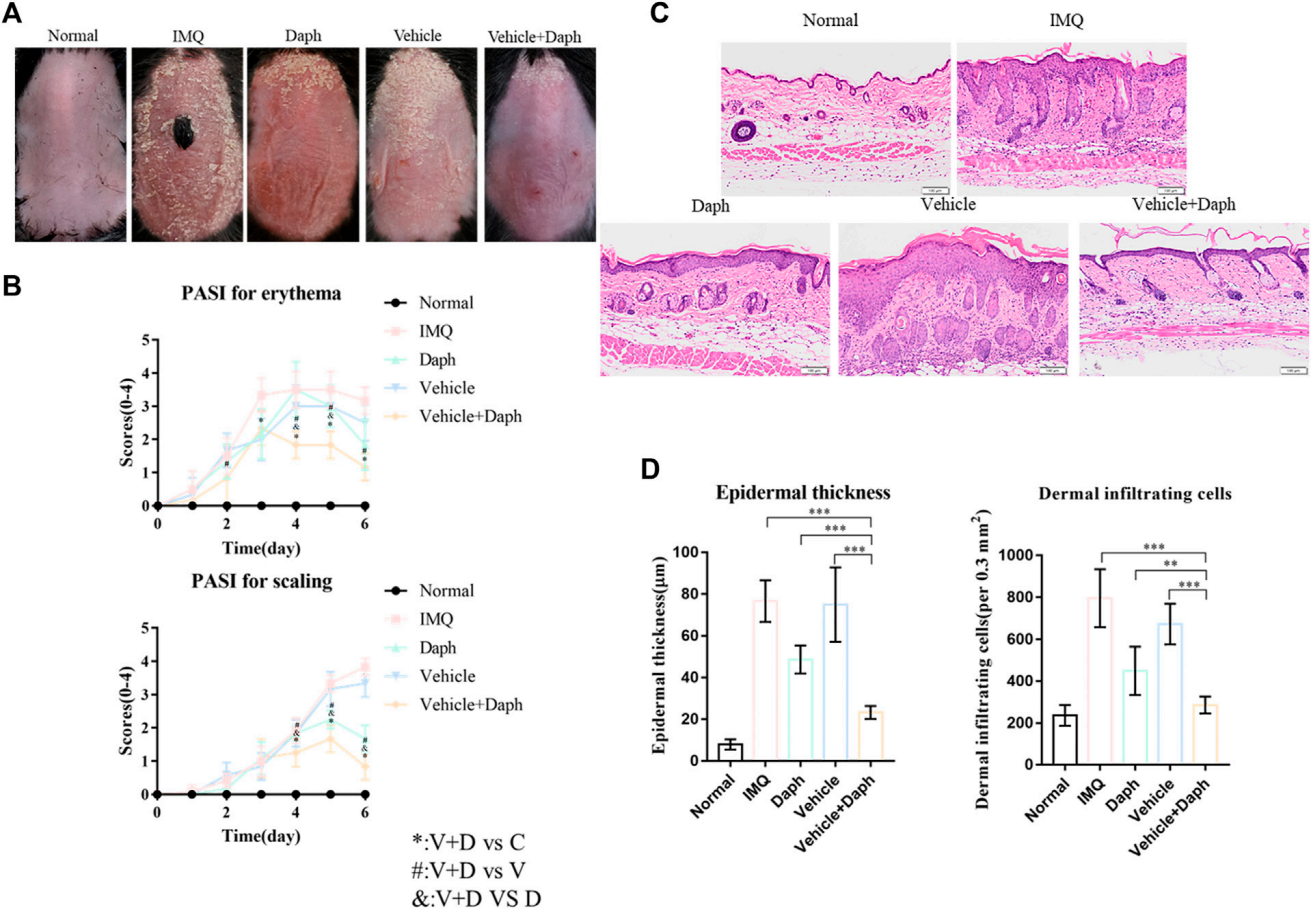
Symptoms and histological results of psoriasis-like mice in different groups. **(A)** Photos of psoriasis modeling sites in different groups of mice. **(B)** H&E staining of psoriasis models in different groups of mice. **(C)** PASI scores of psoriatic mice in different groups. **(D)** Epidermal thickness and the number of inflammatory cells in each group. Data are shown as mean ± SE (*p < 0.05; **p <0.01).

The model of psoriasis-like dermatitis was established in mice with IMQ, and the mice were observed daily and photographed. The modeling of psoriasis-like dermatitis was considered successful according to the photographs. Seven days after the modeling, the mice in the IMQ group showed apparent symptoms of erythema and scales compared with the mice in the control group. Compared with the Daph, vehicle, and IMQ groups, the vehicle + Daph group showed a significant reduction in erythema and the PASI score from day 4 post modeling (Figure 4A,B). The results of HE staining showed that after 7 days of modeling, the symptoms of abnormal epidermal thickening in the vehicle + Daph group were significantly reduced compared with those in other groups (Figure 4C,D).

### 3.4 Daph-Loaded Microneedle Patch Could Inhibit the Abnormal Proliferation of Vγ4+ T Cells and Reduce the Release of Inflammatory Factors of Keratinocytes in Psoriatic Dermatitis

**FIGURE 5:**
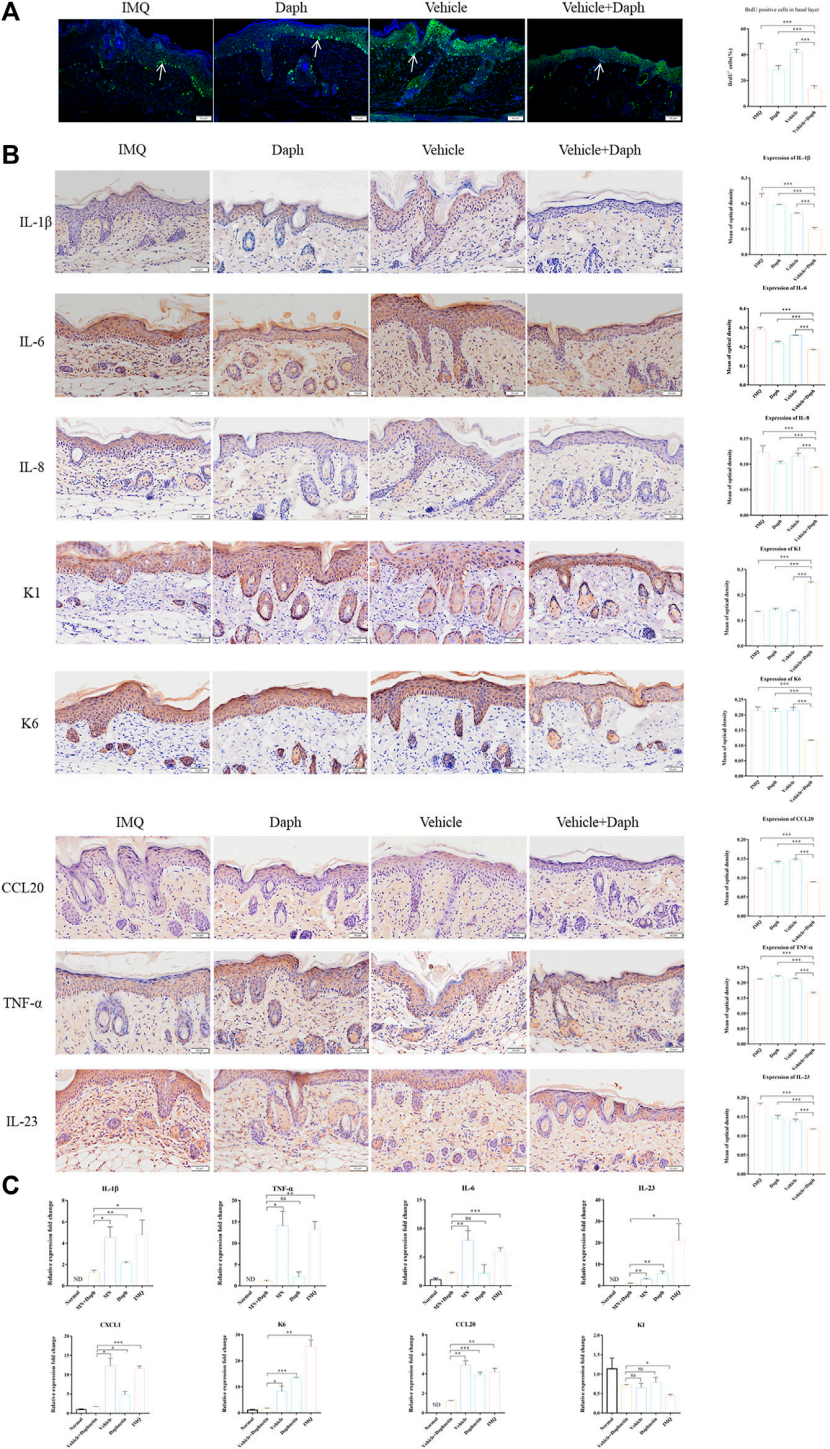
Proliferation ratio of keratinocytes and the secretion of inflammatory factors in psoriatic mice were detected. **(A)** Proportion of keratinocytes in psoriasis modeling sites in each group; DAPI was labeled with blue fluorescence and BrdU was labeled with green fluorescence. **(B)** Immunohistochemical staining of major inflammatory factors (IL-1β, IL-23, TNF- α, IL-6, IL-8, and CCL20) secreted by keratinocytes and the expression of inflammatory factors were counted by the mean optical density. **(C)** qPCR detection results of skin-related inflammatory factors (IL-1β, IL-23, TNF- α, IL-6, CXCL1, and CCL20) at the mouse modeling site. Data are shown as mean ± SE (*p < 0.05; **p < 0.01).

In immunofluorescence staining, BrdU positive cells were in the proliferative stage. The results showed that the abnormal proliferation of keratinocytes in the vehicle + Daph group was significantly lower than that in the other groups (Figure 5A). Immunohistochemical results showed that keratinocyte inflammatory factors IL-8, IL-23, CCL20, and TNF-α decreased significantly in the vehicle + Daph group. The expression of keratinocyte-related marker K6 decreased in the vehicle + Daph group, while the expression of K1 increased (Figure 5B). According to the qPCR result of skin tissue, the expression of inflammatory molecules IL-1β, IL-23, TNF- α, IL-6, CXCL1, and CCL20 in the Daph group was lower than that in the IMQ and vehicle groups, while it was lowest in the vehicle + Daph group. The expression of keratinocyte-related markers K1 and K6 in the MN group was similar to that in the normal group (Figure 5C).

### 3.5 Daph-Loaded Microneedle Patch Inhibited Vγ4+T Cell Recruitment and Inflammatory Secretion

**FIGURE 6:**
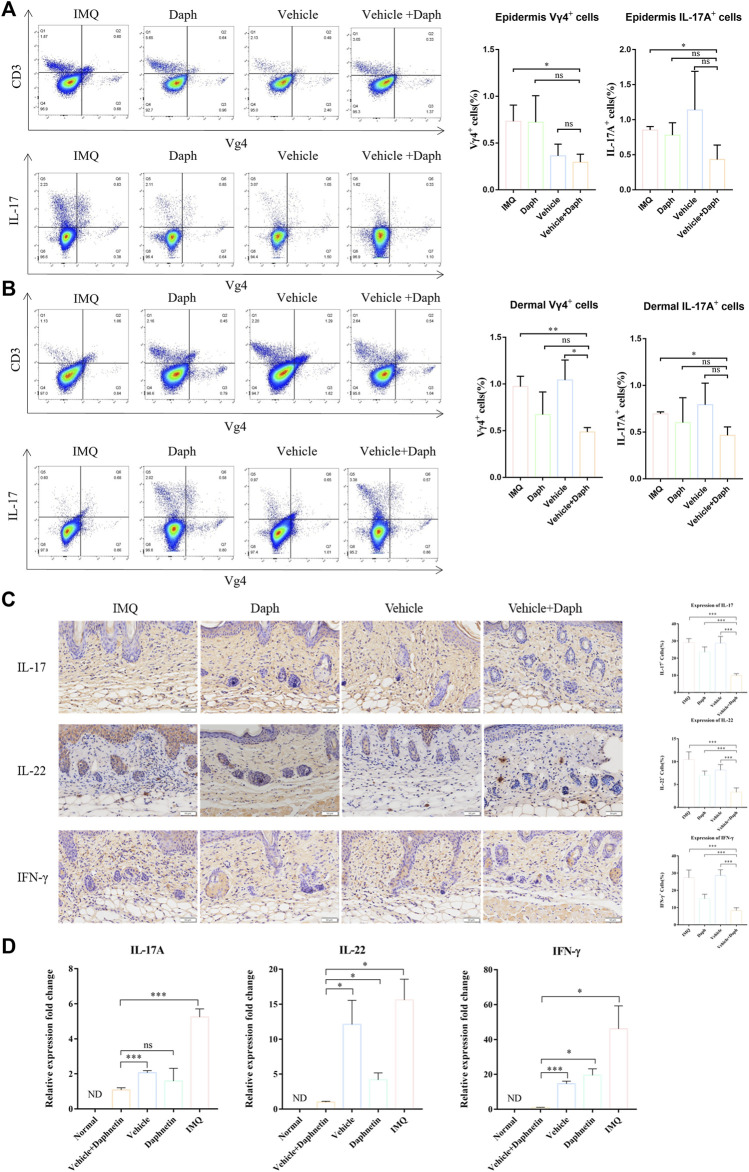
Proportion of Vγ4+T cells and secretion of inflammatory factors in the skin at the modeling site were detected. **(A,B)** Proportion of Vγ4+T cells and the proportion of IL-17-secreting cells in the dermis and epidermis were detected by flow cytometry. **(C)** Immunohistochemical staining of major inflammatory factors (IL-17, IL-22, and IFN-γ) secreted by keratinocytes and the expression of inflammatory factors were counted by the proportion of positive cells. **(D)** PCR detection results of skin-related inflammatory factors (IL-17, IL-22, and IFN-γ) at the mouse modeling site. Data are shown as mean ± SE (*p < 0.05; **p < 0.01).

According to the results of flow cytometry, regardless of the dermis or epidermis, the proportion of Vγ4+T cells in the vehicle + Daph group was lower than that in other groups. In addition, the proportion of IL-17 Vγ4+T secreting cells in the vehicle + Daph group was also the lowest of all groups (Figure 6A,B). The results of immunohistochemical staining showed that the inflammatory factors IL-17, IL-22, and IFN-γ secreted by Vγ4+T cells in the vehicle + Daph group were significantly lower than those in other groups (Figure 6C). These factors were also detected by qPCR at the mRNA level, and the trends were the same as that of immunohistochemistry (Figure 6D).

### 3.6 Daph can Inhibit the Proliferation of HaCaT Cells *In Vitro* but Does Not Affect Apoptosis

**FIGURE 7:**
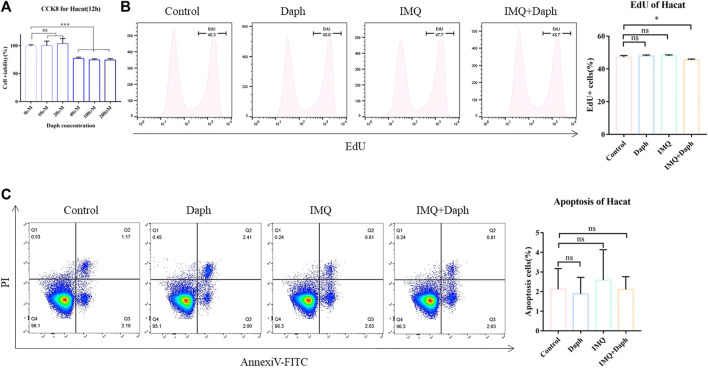
HaCaT cell inflammation model was induced *in vitro* and the effect of Daph on its characteristics was detected. **(A)** CCK-8 was used to detect the effect of different concentrations of Daph on the activity of HaCaT cells. **(B)** Inflammatory state of HaCaT cells was induced by IMQ, and the effect of Daph on its proliferation was detected by an Edu test. **(C)** Effect of Daph on HaCaT cell apoptosis was detected. Data are shown as mean ± SE (*p < 0.05; **p < 0.01).

Daph with a gradient concentration was added to the HaCaT cell culture medium. The CCK-8 assay showed that when the concentration of Daph was not more than 20 μM, it exhibited no cytotoxicity to normal HaCaT cells (Figure 7A). Therefore, 20 μM was selected as the Daph concentration *in vitro*. The inflammatory response of HaCaT cells was induced by IMQ *in vitro*. Edu results showed that Daph could inhibit the proliferation of HaCaT cells in an inflammatory environment (Figure 7B) but had no significant effect on apoptosis (Figure 7C).

### 3.7 Daph can Inhibit the Expression of Inflammatory Factors and the NF-κB Signaling Pathway in HaCaT Cells *in vitro*

**FIGURE 8:**
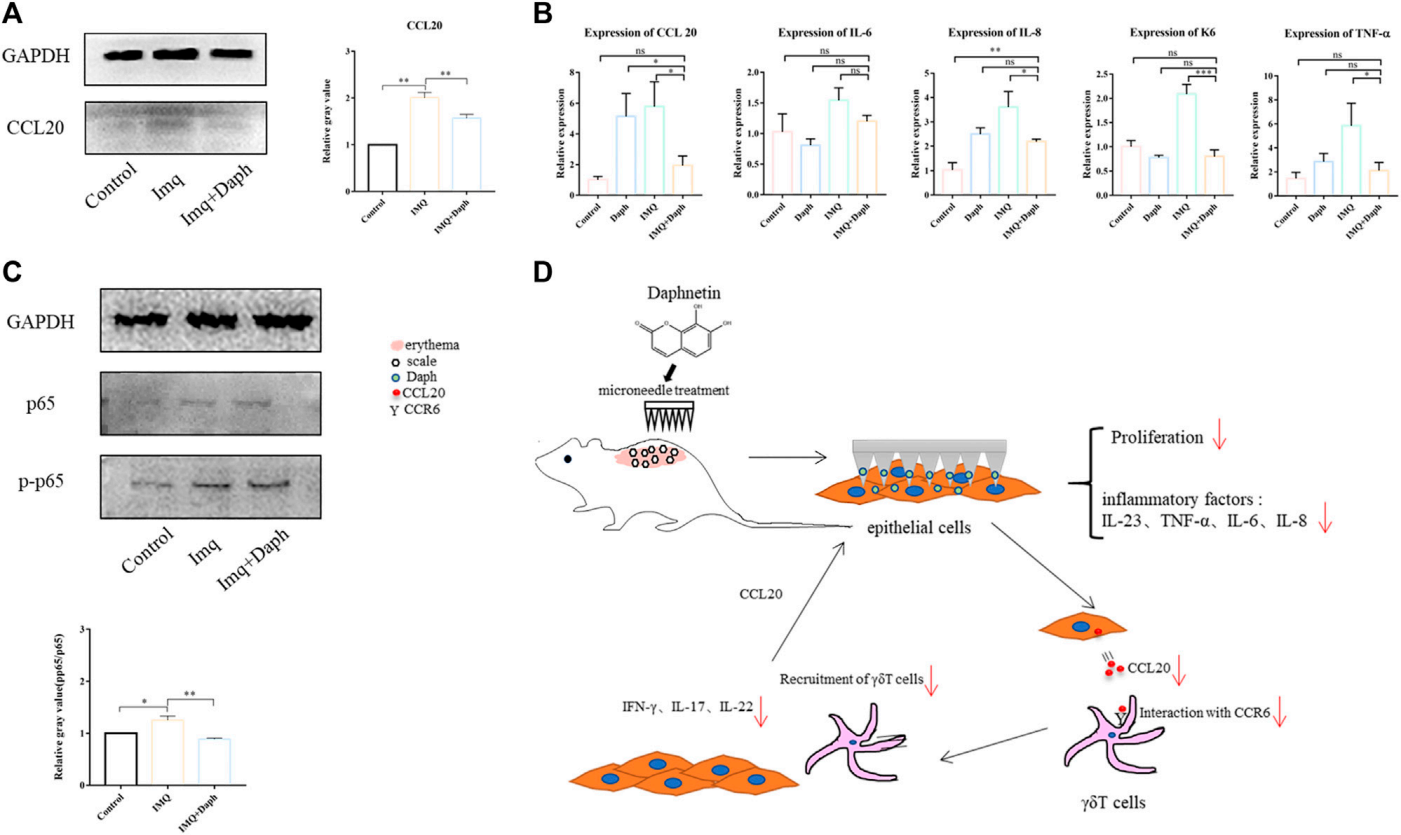
Effects of Daph on the secretion of inflammatory factors and activation of the NF-κB signaling pathway in HaCaT cells were detected. **(A)** Effect of Daph on CCL20 secretion of HaCaT cells in the inflammatory state was detected by WB. **(B)** Effect of Daph on major inflammatory factors in HaCaT cells was detected by qPCR. **(C)** Effect of Daph on the activation of the NF-κB signaling pathway in HaCaT cells was detected by WB. **(D)** Schematic diagram of the mechanism of using MN-loaded Daph to treat psoriasis. Data are shown as mean ± SE (*p < 0.05; **p < 0.01).

The expression of CCL20 in HaCaT cells under an inflammatory environment was detected with Western blot. The results showed that after treating with Daph, the expression of CCL20 in HaCaT cells was downregulated (Figure 8A). IL-6, IL-8, CCL20, and TNF-α were detected at the mRNA level by qPCR. The results showed that the expression of inflammatory factors in HaCaT cells was inhibited after adding Daph (Figure 8B). One hour after adding Daph, the key signal molecule p65 of NF-κB was detected. The results showed that Daph could inhibit the phosphorylation of p65 and inhibit the NF-κB signaling pathway (Figure 8C).

## 4 Discussion

The treatment of psoriasis is a challenge because of its pathogenic complexity (Greb et al., 2016; Dainichi et al., 2018). Some potential drugs and methods for the treatment of psoriasis were proposed in some studies recently (Hawkes et al., 2017), among which Daph showed a good symptom relief effect in the mouse psoriasis model (Gao et al., 2020). However, during the onset of psoriasis, Daph does not penetrate the thickened epidermis readily, making it difficult to reach the subcutaneous layer. As Daph is an unstable drug, it degrades rapidly, and thus does not maintain its therapeutic effect. Therefore, we chose the soluble MN to carry Daph and added the CNC to the MN for better mechanical properties. The MN can be used for transdermal administration as an effective alternative to traditional administration (Ahmed Saeed Al-Japairai et al., 2020). The inverted pyramid tip of modified MNs showed better mechanical properties and larger skin contact areas than the conical tip so that the MN could penetrate the skin better and enhance the subcutaneous drug release. In addition, the modified MN is well-degradable *in vivo* and can deliver the drug to 200 μm in depth, which meets the demand for drug delivery *in vivo*.

The main symptoms of psoriasis are epidermal thickening, severe erythema, and scales (2). In the early stage of psoriasis, after being activated by various external factors, dendritic cells and keratinocytes start to show innate immune response through the secretion of innate immune molecules such as AMP (Mahil et al., 2016) and then induce the activation of T cells in the skin to secrete pro-inflammatory factors such as IL-17 and IL-22 (Cai et al., 2011). Recent studies have shown that the inhibition of the IL-23/IL-17 inflammatory axis plays an important role in the treatment and symptom relief of psoriasis (Ni and Lai, 2020). These pro-inflammatory factors stimulate the abnormal proliferation of keratinocytes and secrete a large number of chemokines and antimicrobial peptides, which could recruit more immune cells (Li et al., 2018). Inflammatory cells infiltrate the skin and form a positive inflammatory loop to mediate the occurrence and development of psoriasis, among which CCL20 plays a key role ((Homey et al., 2000), (Harper et al., 2009), (Furue et al., 2020b)). CCL20 secreted by keratinocytes induces CCR6-expressing T cells to infiltrate the skin and secrete a related inflammatory shadow, which promotes the further proliferation and differentiation of keratinocytes and amplifies this response (Shibata et al., 2015). Therefore, CCL20 has also been studied as a possible therapeutic target for psoriasis (Mabuchi et al., 2013; Getschman et al., 2017; Li et al., 2020). In this study, IMQ was used for the construction of a psoriasis-like dermatitis model in mice for its TLR7/8 activation effect (Gilliet et al., 2004). Using IMQ to construct the psoriasis model in mice is a mature disease model construction method (van der Fits et al., 2009; Swindell et al., 2017). After using an MN patch loading Daph, the symptoms of psoriasis-like dermatitis in mice were significantly relieved. In addition to the relieved symptoms, the production of various inflammatory factors, especially CCL20, was significantly reduced.

To identify the mechanism by which Daph reduces CCL20 secretion by keratinocytes, we used HaCaT cells for experimental verification *in vitro*. According to previous studies, Daph is an inhibitor of the NF-κB signaling pathway (Lv et al., 2018), and the NF-κB pathway is the key to regulating the production of CCL20 ((Shin et al., 2020), (Yu et al., 2021)). We used IMQ to construct the HaCaT cell inflammation model *in vitro*, added Daph as a stimulus, and then detected the associated biomarkers. As expected, after adding Daph, the NF-κB signaling pathway of HaCaT cells was inhibited and the expression of CCL20 decreased. In addition, the expression of other inflammatory factors also decreased, such as IL-8 and TNF-α. The *in vivo* results showed that the proportion of Vγ4+ T cells chemotaxed by CCL20 decreased significantly after treatment with MN patches loaded with Daph. IL-17, IL-22, and IFN-γ secreted by Vγ4+ T cells can again affect keratinocytes producing more inflammatory factors to form a positive inflammatory feedback loop (Bata-Csorgo et al., 1995). Therefore, we believe that the therapeutic effect of Daph in psoriasis relies on the inhibition of keratinocyte proliferation and the inhibition of CCL20, which disrupts the inflammatory loop of psoriasis. However, although the production of CCL20 is mainly regulated by the NF-κB pathway, it is also affected by P38/ERK/JNK and RIP4/STAT3 signaling pathways (Bae et al., 2018; Furue et al., 2019; Furue et al., 2020c). Whether Daph regulates these pathways remains to be further studied. However, Daph as a therapeutic drug for psoriasis is still in the experimental stage, and its therapeutic mechanism and safety need to be more perfect with clinical verification.

This study revealed that the modified hyaluronic acid MN, as a vehicle of drug administration, delivers the drug more effectively than having the drug applied alone topically in psoriasis. In addition, the mechanism of Daph in the treatment of psoriasis and inhibition of inflammatory response has been studied. Therefore, the MN patch used in this study can be ubiquitously applied in the research of psoriasis treatment. Daph and other drugs can be used in combination to obtain better therapeutic results.

## 5 Conclusion

In this research, we modified the hyaluronic acid MN for better morphology and mechanical properties. The modified MN was optimum for transdermal administration, and the symptoms of psoriatic mice were significantly alleviated by applying a Daph-loaded MN. *In vitro*, Daph can also inhibit the proliferation of HaCaT cells in the inflammatory state. Further studies showed that Daph could inhibit the NF-κB pathway, which was the key signaling pathway controlling the production of CCL20, an important inflammatory factor in terms of the pathogenesis of psoriasis. The use of modified MNs and the identification of a new mechanism of Daph in psoriasis treatment provide a new research direction for the psoriasis study (Onderdijk et al., 2017).

## Data Availability

The raw data supporting the conclusions of this article will be made available by the authors, without undue reservation.

## Supplementary Material

Supplementary Figure S1 Schematic illustration showing the fabricating process of HA/PVA/CNC/Daph microneedle patches.

## References

[B1] Ahmed Saeed Al-Japairai K. Mahmood S. Hamed Almurisi S. Reddy Venugopal J. Rebhi Hilles A. Azmana M. (2020). Current Trends in Polymer Microneedle for Transdermal Drug Delivery. Int. J. Pharm. 587, 119673. 10.1016/j.ijpharm.2020.119673 PubMed Abstract | 10.1016/j.ijpharm.2020.119673 | Google Scholar 32739388 PMC7392082

[B2] Albanesi C. Madonna S. Gisondi P. Girolomoni G. (2018). The Interplay between Keratinocytes and Immune Cells in the Pathogenesis of Psoriasis. Front. Immunol. 9, 1549. 10.3389/fimmu.2018.01549 PubMed Abstract | 10.3389/fimmu.2018.01549 | Google Scholar 30034395 PMC6043636

[B3] Armstrong A. W. Read C. (2020). Pathophysiology, Clinical Presentation, and Treatment of Psoriasis. JAMA 323 (19), 1945–1960. 10.1001/jama.2020.4006 PubMed Abstract | 10.1001/jama.2020.4006 | Google Scholar 32427307

[B4] Bae H. C. Jeong S. H. Kim J. H. Lee H. Ryu W.-I. Kim M.-G. (2018). RIP4 Upregulates CCL20 Expression through STAT3 Signalling in Cultured Keratinocytes. Exp. Dermatol 27 (10), 1126–1133. 10.1111/exd.13750 PubMed Abstract | 10.1111/exd.13750 | Google Scholar 30044012

[B5] Bata-Csorgo Z. Hammerberg C. Voorhees J. J. Cooper K. D. (1995). Intralesional T-Lymphocyte Activation as a Mediator of Psoriatic Epidermal Hyperplasia. J. Invest. Dermatol 105, 89S–94S. 10.1111/1523-1747.ep12316121 PubMed Abstract | 10.1111/1523-1747.ep12316121 | Google Scholar 7616005

[B6] Cai Y. Shen X. Ding C. Qi C. Li K. Li X. (2011). Pivotal Role of Dermal IL-17-Producing γδ T Cells in Skin Inflammation. Immunity 35 (4), 596–610. 10.1016/j.immuni.2011.08.001 PubMed Abstract | 10.1016/j.immuni.2011.08.001 | Google Scholar 21982596 PMC3205267

[B7] Dainichi T. Kitoh A. Otsuka A. Nakajima S. Nomura T. Kaplan D. H. (2018). The Epithelial Immune Microenvironment (EIME) in Atopic Dermatitis and Psoriasis. Nat. Immunol. 19 (12), 1286–1298. 10.1038/s41590-018-0256-2 PubMed Abstract | 10.1038/s41590-018-0256-2 | Google Scholar 30446754

[B8] Du H. Liu P. Zhu J. Lan J. Li Y. Zhang L. (2019). Hyaluronic Acid-Based Dissolving Microneedle Patch Loaded with Methotrexate for Improved Treatment of Psoriasis. ACS Appl. Mat. Interfaces 11 (46), 43588–43598. 10.1021/acsami.9b15668 PubMed Abstract | 10.1021/acsami.9b15668 | Google Scholar 31651148

[B9] Furue K. Ito T. Tanaka Y. Hashimoto-Hachiya A. Takemura M. Murata M. (2020). The EGFR-ERK/JNK-CCL20 Pathway in Scratched Keratinocytes May Underpin Koebnerization in Psoriasis Patients. Int. J. Mol. Sci. 21 (2), 434. 10.3390/ijms21020434 PubMed Abstract | 10.3390/ijms21020434 | Google Scholar PMC7013594 31936670

[B10] Furue K. Ito T. Tsuji G. Nakahara T. Furue M. (2020). The CCL20 and CCR6 axis in Psoriasis. Scand. J. Immunol. 91 (3), e12846. 10.1111/sji.12846 PubMed Abstract | 10.1111/sji.12846 | Google Scholar 31692008

[B11] Furue K. Ito T. Tanaka Y. Yumine A. Hashimoto-Hachiya A. Takemura M. (2019). Cyto/chemokine Profile of *In Vitro* Scratched Keratinocyte Model: Implications of Significant Upregulation of CCL20, CXCL8 and IL36G in Koebner Phenomenon. J. Dermatological Sci. 94 (1), 244–251. 10.1016/j.jdermsci.2019.04.002 PubMed Abstract | 10.1016/j.jdermsci.2019.04.002 | Google Scholar 31010609

[B12] Furue M. Furue K. Tsuji G. Nakahara T. (2020). Interleukin-17A and Keratinocytes in Psoriasis. Int. J. Mol. Sci. 21 (4), 1275. 10.3390/ijms21041275 PubMed Abstract | 10.3390/ijms21041275 | Google Scholar PMC7072868 32070069

[B13] Gao J. Chen F. Fang H. Mi J. Qi Q. Yang M. (2020). Daphnetin Inhibits Proliferation and Inflammatory Response in Human HaCaT Keratinocytes and Ameliorates Imiquimod-Induced Psoriasis-like Skin Lesion in Mice. Biol. Res. 53 (1), 48. 10.1186/s40659-020-00316-0 PubMed Abstract | 10.1186/s40659-020-00316-0 | Google Scholar 33081840 PMC7576854

[B14] Georgescu S. R. Tampa M. Caruntu C. Sarbu M. I. Mitran C. I. Mitran M. I. (2019). Advances in Understanding the Immunological Pathways in Psoriasis. Int. J. Mol. Sci. 20 (3), 739. 10.3390/ijms20030739 PubMed Abstract | 10.3390/ijms20030739 | Google Scholar PMC6387410 30744173

[B15] Getschman A. E. Imai Y. Larsen O. Peterson F. C. Wu X. Rosenkilde M. M. (2017). Protein Engineering of the Chemokine CCL20 Prevents Psoriasiform Dermatitis in an IL-23-dependent Murine Model. Proc. Natl. Acad. Sci. U.S.A. 114 (47), 12460–12465. 10.1073/pnas.1704958114 PubMed Abstract | 10.1073/pnas.1704958114 | Google Scholar 29109267 PMC5703275

[B16] Gilliet M. Conrad C. Geiges M. Cozzio A. Thürlimann W. Burg G. (2004). Psoriasis Triggered by Toll-like Receptor 7 Agonist Imiquimod in the Presence of Dermal Plasmacytoid Dendritic Cell Precursors. Arch. Dermatol 140 (12), 1490–1495. 10.1001/archderm.140.12.1490 PubMed Abstract | 10.1001/archderm.140.12.1490 | Google Scholar 15611427

[B17] Greb J. E. Goldminz A. M. Elder J. T. Lebwohl M. G. Gladman D. D. Wu J. J. (2016). Psoriasis. Nat. Rev. Dis. Prim. 2, 16082. 10.1038/nrdp.2016.82 PubMed Abstract | 10.1038/nrdp.2016.82 | Google Scholar 27883001

[B18] Harper E. G. Guo C. Rizzo H. Lillis J. V. Kurtz S. E. Skorcheva I. (2009). Th17 Cytokines Stimulate CCL20 Expression in Keratinocytes *In Vitro* and *In Vivo*: Implications for Psoriasis Pathogenesis. J. Investigative Dermatology 129 (9), 2175–2183. 10.1038/jid.2009.65 PubMed Abstract | 10.1038/jid.2009.65 | Google Scholar PMC2892172 19295614

[B19] Hawkes J. E. Chan T. C. Krueger J. G. (2017). Psoriasis Pathogenesis and the Development of Novel Targeted Immune Therapies. J. Allergy Clin. Immunol. 140 (3), 645–653. 10.1016/j.jaci.2017.07.004 PubMed Abstract | 10.1016/j.jaci.2017.07.004 | Google Scholar 28887948 PMC5600287

[B20] Homey B. Dieu-Nosjean M.-C. Wiesenborn A. Massacrier C. Pin J.-J. Oldham E. (2000). Up-Regulation of Macrophage Inflammatory Protein-3α/CCL20 and CC Chemokine Receptor 6 in Psoriasis. J. Immunol. 164 (12), 6621–6632. 10.4049/jimmunol.164.12.6621 PubMed Abstract | 10.4049/jimmunol.164.12.6621 | Google Scholar 10843722

[B21] Huang S. Liu H. Huang S. Fu T. Xue W. Guo R. (2020). Dextran Methacrylate Hydrogel Microneedles Loaded with Doxorubicin and Trametinib for Continuous Transdermal Administration of Melanoma. Carbohydr. Polym. 246, 116650. 10.1016/j.carbpol.2020.116650 PubMed Abstract | 10.1016/j.carbpol.2020.116650 | Google Scholar 32747282

[B22] Kim N. Lee S. Kang J. Choi Y. A. Jang Y. H. Jeong G. S. (2021). Cudraxanthone D Ameliorates Psoriasis-like Skin Inflammation in an Imiquimod-Induced Mouse Model via Inhibiting the Inflammatory Signaling Pathways. Molecules 26 (19), 6086. 10.3390/molecules26196086 PubMed Abstract | 10.3390/molecules26196086 | Google Scholar 34641629 PMC8512696

[B23] Li H.-J. Wu N.-L. Pu C.-M. Hsiao C.-Y. Chang D.-C. Hung C.-F. (2020). Chrysin Alleviates Imiquimod-Induced Psoriasis-like Skin Inflammation and Reduces the Release of CCL20 and Antimicrobial Peptides. Sci. Rep. 10 (1), 2932. 10.1038/s41598-020-60050-1 PubMed Abstract | 10.1038/s41598-020-60050-1 | Google Scholar 32076123 PMC7031269

[B24] Li Y. Wu J. Luo G. He W. (2018). Functions of Vγ4 T Cells and Dendritic Epidermal T Cells on Skin Wound Healing. Front. Immunol. 9, 1099. 10.3389/fimmu.2018.01099 PubMed Abstract | 10.3389/fimmu.2018.01099 | Google Scholar 29915573 PMC5994537

[B25] Liu T. Luo G. Xing M. (2020). Biomedical Applications of Polymeric Microneedles for Transdermal Therapeutic Delivery and Diagnosis: Current Status and Future Perspectives. Adv. Ther. 3 (9), 1900140. 10.1002/adtp.201900140 10.1002/adtp.201900140 | Google Scholar

[B26] Lowes M. A. Suárez-Fariñas M. Krueger J. G. (2014). Immunology of Psoriasis. Annu. Rev. Immunol. 32, 227–255. 10.1146/annurev-immunol-032713-120225 PubMed Abstract | 10.1146/annurev-immunol-032713-120225 | Google Scholar 24655295 PMC4229247

[B27] Lv H. Fan X. Wang L. Feng H. Ci X. (2018). Daphnetin Alleviates Lipopolysaccharide/d-Galactosamine-Induced Acute Liver Failure via the Inhibition of NLRP3, MAPK and NF-Κb, and the Induction of Autophagy. Int. J. Biol. Macromol. 119, 240–248. 10.1016/j.ijbiomac.2018.07.101 PubMed Abstract | 10.1016/j.ijbiomac.2018.07.101 | Google Scholar 30031824

[B28] Mabuchi T. Singh T. P. Takekoshi T. Jia G.-f. Wu X. Kao M. C. (2013). CCR6 Is Required for Epidermal Trafficking of γδ-T Cells in an IL-23-Induced Model of Psoriasiform Dermatitis. J. Investigative Dermatology 133 (1), 164–171. 10.1038/jid.2012.260 10.1038/jid.2012.260 | Google Scholar PMC3511632 22895364

[B29] Mahil S. K. Capon F. Barker J. N. (2016). Update on Psoriasis Immunopathogenesis and Targeted Immunotherapy. Semin. Immunopathol. 38 (1), 11–27. 10.1007/s00281-015-0539-8 PubMed Abstract | 10.1007/s00281-015-0539-8 | Google Scholar 26573299 PMC4706579

[B30] Nestle F. O. Kaplan D. H. Barker J. (2009). Psoriasis. N. Engl. J. Med. 361 (5), 496–509. 10.1056/nejmra0804595 PubMed Abstract | 10.1056/nejmra0804595 | Google Scholar 19641206

[B31] Ni X. Lai Y. (2020). Keratinocyte: A Trigger or an Executor of Psoriasis? J. Leukoc. Biol. 108 (2), 485–491. 10.1002/jlb.5mr0120-439r PubMed Abstract | 10.1002/jlb.5mr0120-439r | Google Scholar 32170886

[B32] Onderdijk A. J. Hekking-Weijma I. M. Florencia E. F. Prens E. P. (2017). Surgical Denervation in the Imiquimod-Induced Psoriasiform Mouse Model. Methods Mol. Biol. 1559, 75–81. 10.1007/978-1-4939-6786-5_6 PubMed Abstract | 10.1007/978-1-4939-6786-5_6 | Google Scholar 28063038

[B33] Pei Q. Hu P. Zhang H. Li H. Yang T. Liu R. (2021). Daphnetin Exerts an Anticancer Effect by Attenuating the Pro-inflammatory Cytokines. J. Biochem. Mol. Toxicol. 35 (6), 1–8. 10.1002/jbt.22759 10.1002/jbt.22759 | Google Scholar 33749080

[B34] Shibata S. Tada Y. Hau C. S. Mitsui A. Kamata M. Asano Y. (2015). Adiponectin Regulates Psoriasiform Skin Inflammation by Suppressing IL-17 Production from γδ-T Cells. Nat. Commun. 6, 7687. 10.1038/ncomms8687 PubMed Abstract | 10.1038/ncomms8687 | Google Scholar 26173479

[B35] Shin J.-W. Kwon M.-a. Hwang J. Lee S.-J. Lee J.-H. Kim H.-J. (2020). Keratinocyte Transglutaminase 2 Promotes CCR6+ γδT-cell Recruitment by Upregulating CCL20 in Psoriatic Inflammation. Cell Death Dis. 11 (4), 301. 10.1038/s41419-020-2495-z PubMed Abstract | 10.1038/s41419-020-2495-z | Google Scholar 32355189 PMC7193648

[B36] Swindell W. R. Michaels K. A. Sutter A. J. Diaconu D. Fritz Y. Xing X. (2017). Imiquimod Has Strain-dependent Effects in Mice and Does Not Uniquely Model Human Psoriasis. Genome Med. 9 (1), 24. 10.1186/s13073-017-0415-3 PubMed Abstract | 10.1186/s13073-017-0415-3 | Google Scholar 28279190 PMC5345243

[B37] van der Fits L. Mourits S. Voerman J. S. A. Kant M. Boon L. Laman J. D. (2009). Imiquimod-induced Psoriasis-like Skin Inflammation in Mice Is Mediated via the IL-23/IL-17 axis. J. Immunol. 182 (9), 5836–5845. 10.4049/jimmunol.0802999 PubMed Abstract | 10.4049/jimmunol.0802999 | Google Scholar 19380832

[B38] Vicic M. Kastelan M. Brajac I. Sotosek V. Massari L. P. (2021). Current Concepts of Psoriasis Immunopathogenesis. Int. J. Mol. Sci. 22 (21), 1154. 10.3390/ijms222111574 PubMed Abstract | 10.3390/ijms222111574 | Google Scholar 34769005 PMC8584028

[B39] Weiss G. Shemer A. Trau H. (2002). The Koebner Phenomenon: Review of the Literature. J. Eur. Acad. Dermatology Venereol. JEADV. 16 (3), 241–248. 10.1046/j.1473-2165.2002.00406.x PubMed Abstract | 10.1046/j.1473-2165.2002.00406.x | Google Scholar 12195563

[B40] Yu Y. Xue X. Tang W. Su L. Zhang L. Zhang Y. (2021). Cytosolic-DNA-mediated STING-dependent Inflammation Contributes to the Progression of Psoriasis. J. investigative dermatology. 142. 898. 10.1016/j.jid.2021.08.430 10.1016/j.jid.2021.08.430 | Google Scholar 34537189

[B41] Zhu R. Cai X. Zhou C. Li Y. Zhang X. Li Y. (2017). Dermal Vγ4+T Cells Enhance the IMQ-Induced Psoriasis-like Skin Inflammatidon in Re-challenged Mice. Am. J. Transl. Res. 9 (12), 5347–5360. PubMed Abstract | Google Scholar 29312488 PMC5752886

