# Supplementary figures and images for: A Modified Hyaluronic Acid–Based Dissolving Microneedle Loaded With Daphnetin Improved the Treatment of Psoriasis
Source: Front Bioeng Biotechnol. 2022 Jun 17;10:900274. doi: 10.3389/fbioe.2022.900274 (PMC9367187; doi:10.3389/fbioe.2022.900274)

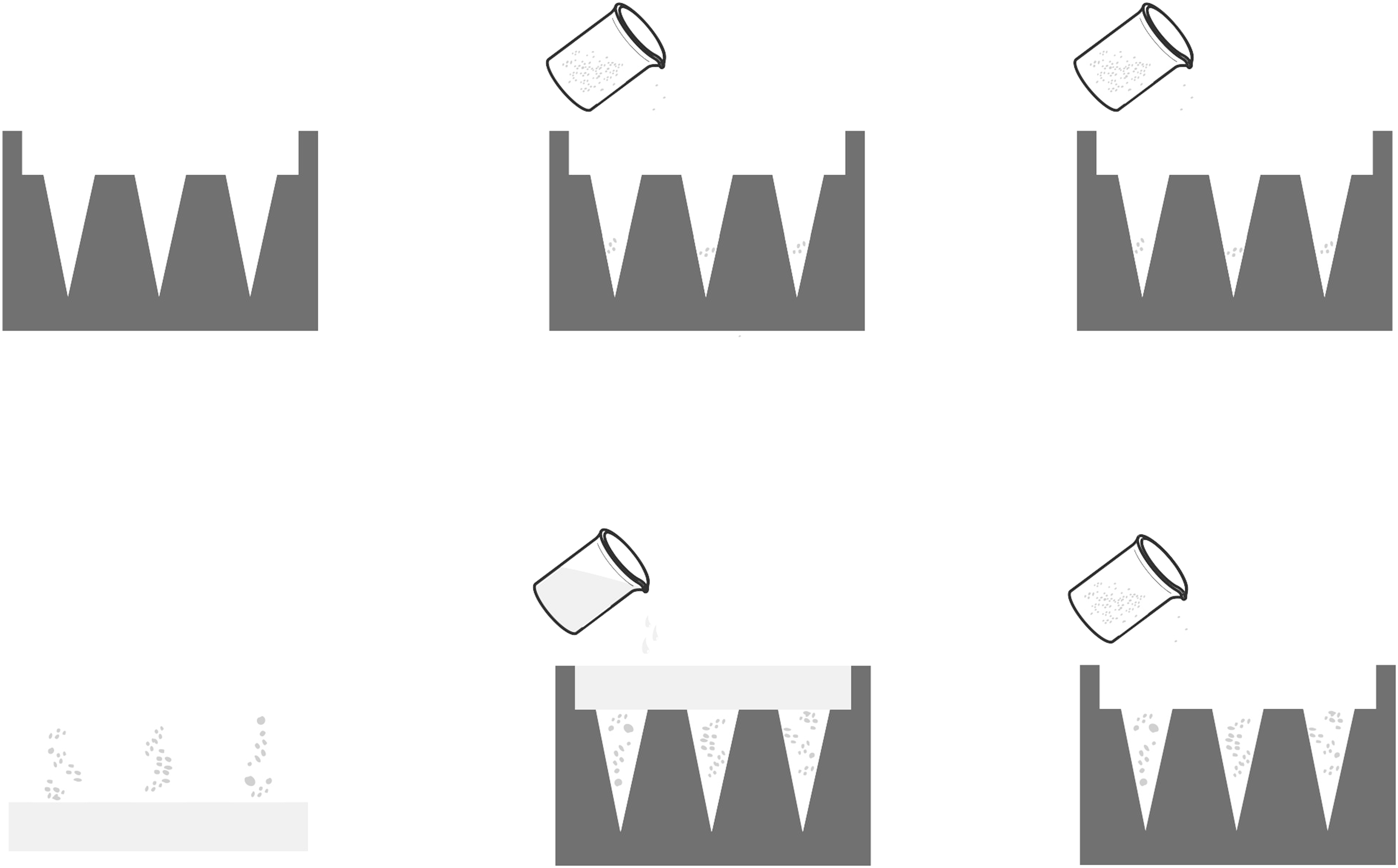

Supplement: Supplementary file 1 [file Image1.tif]
